# Supplementary material for: Patterns and prognosis of holding regimens for people living with HIV in Asian countries
Source: PLoS One. 2022 Mar 30;17(3):e0264157. doi: 10.1371/journal.pone.0264157 (PMC8967045; doi:10.1371/journal.pone.0264157)
Supplement: S1 Table — 3TC, lamivudine; ABC, abacavir; ATV, atazanavir; AZT, zidovudine; COB, cobicistat; D4T, stavudine; DDC, zalcitabine; DDI, didanosine; DRV, darunavir; EFV, efavirenz; ETV, etravirine; EVG, elvitegravir; FAP, fosamprenavir; FTC, emtricitabine; HYD, hydroxyurea; IDV, indinavir; IL2, interleukin-2; LPV, lopinavir; MVC, maraviroc; NFV, nelfinavir; NNRTI, non-nucleoside reverse-transcriptase inhibitor; NRTI, nucleoside reverse transcriptase inhibitor; NVP, nevirapine; PI, protease inhibitor; RAL, raltegravir; RCT, randomized controlled trial; RIT, ritonavir; RPV, rilpivirine; RTF, ritonavir full dose; SQF, saquinavir fortovase; SQI, saquinavir invirase; TDF, tenofovir disoproxil fumarate. (DOCX) [file pone.0264157.s001.docx]

**S1 Table. Detailed patterns of holding regimens**

| **Patterns** | **Frequency** | **Percent** |
| --- | --- | --- |
| **NRTI + NNRTI** | **188** | **44.2** |
| 3TC+AZT+EFV | 46 | 10.82 |
| 3TC+AZT+NVP | 37 | 8.71 |
| 3TC+EFV+TDF | 29 | 6.82 |
| 3TC+D4T+NVP | 18 | 4.24 |
| 3TC+D4T+EFV | 15 | 3.53 |
| DDI+D4T+EFV | 8 | 1.88 |
| 3TC+NVP+TDF | 7 | 1.65 |
| ABC+3TC+NVP | 7 | 1.65 |
| ABC+3TC+EFV | 4 | 0.94 |
| DDI+AZT+EFV | 3 | 0.71 |
| DDI+D4T+NVP | 3 | 0.71 |
| FTC+EFV+TDF | 3 | 0.71 |
| 3TC+RPV+TDF | 2 | 0.47 |
| DDI+3TC+EFV | 2 | 0.47 |
| FTC+NVP+TDF | 2 | 0.47 |
| ABC+3TC+RPV | 1 | 0.24 |
| DDI+D4T+HYD+NVP | 1 | 0.24 |
| **NRTI + PI** | **189** | **44.5** |
| 3TC+ATV+RIT+TDF | 33 | 7.76 |
| 3TC+RIT+LPV+TDF | 16 | 3.76 |
| FTC+RIT+LPV+TDF | 16 | 3.76 |
| 3TC+AZT+IDV | 13 | 3.06 |
| 3TC+AZT+RIT+LPV | 12 | 2.82 |
| 3TC+AZT+ATV+RIT | 11 | 2.59 |
| 3TC+D4T+RIT+LPV | 6 | 1.41 |
| ABC+3TC+ATV+RIT | 6 | 1.41 |
| ABC+3TC+RIT+LPV | 6 | 1.41 |
| FTC+ATV+RIT+TDF | 6 | 1.41 |
| 3TC+ATV+TDF | 5 | 1.18 |
| 3TC+D4T+IDV | 5 | 1.18 |
| DDI+3TC+ATV | 4 | 0.94 |
| DDI+3TC+IDV | 4 | 0.94 |
| FTC+RIT+DRV+TDF | 4 | 0.94 |
| ABC+3TC+RIT+DRV | 3 | 0.71 |
| AZT+RIT+LPV+TDF | 3 | 0.71 |
| DDI+3TC+RIT+LPV | 3 | 0.71 |
| 3TC+AZT+ATV | 2 | 0.47 |
| 3TC+AZT+ATV+RIT+TDF | 2 | 0.47 |
| 3TC+AZT+IDV+RIT | 2 | 0.47 |
| 3TC+AZT+NFV | 2 | 0.47 |
| AZT+FTC+ATV+RIT+TDF | 2 | 0.47 |
| DDI+3TC+NFV | 2 | 0.47 |
| DDI+AZT+IDV+RIT | 2 | 0.47 |
| 3TC+ATV+RIT | 1 | 0.24 |
| 3TC+D4T+IDV+RIT | 1 | 0.24 |
| 3TC+D4T+NFV | 1 | 0.24 |
| 3TC+FAP+TDF | 1 | 0.24 |
| 3TC+NFV+TDF | 1 | 0.24 |
| ABC+3TC+D4T+RIT+LPV | 1 | 0.24 |
| ABC+3TC+NFV+RIT | 1 | 0.24 |
| ABC+D4T+RIT+LPV | 1 | 0.24 |
| AZT+ATV+RIT+TDF | 1 | 0.24 |
| AZT+RIT+DRV+TDF | 1 | 0.24 |
| DDC+AZT+IDV | 1 | 0.24 |
| DDI+3TC+ATV+RIT | 1 | 0.24 |
| DDI+3TC+IDV+RIT | 1 | 0.24 |
| DDI+D4T+RIT+LPV | 1 | 0.24 |
| FTC+ATV+RIT | 1 | 0.24 |
| FTC+ATV+TDF | 1 | 0.24 |
| 3TC+AZT+RIT+SQI | 1 | 0.24 |
| D4T+RTF+SQF | 1 | 0.24 |
| DDI+AZT+RIT+SQI | 1 | 0.24 |
| **Other combination** | **48** | **11.3** |
| RAL+RIT+LPV | 7 | 1.65 |
| RCT | 6 | 1.41 |
| 3TC+RAL+TDF | 3 | 0.71 |
| 3TC+AZT+RAL | 2 | 0.47 |
| 3TC+D4T | 2 | 0.47 |
| 3TC+TDF | 2 | 0.47 |
| DDI+D4T | 2 | 0.47 |
| EFV+IDV | 2 | 0.47 |
| FTC+COB+TDF+EVG | 2 | 0.47 |
| 3TC+AZT+ETV+RIT+DRV | 1 | 0.24 |
| 3TC+EFV+RIT+LPV | 1 | 0.24 |
| 3TC+NVP+IDV+RIT | 1 | 0.24 |
| 3TC+RAL+ATV+RIT | 1 | 0.24 |
| 3TC+RAL+RIT+LPV | 1 | 0.24 |
| ABC+DDI+3TC | 1 | 0.24 |
| D4T+NVP+RIT+LPV | 1 | 0.24 |
| DDI+3TC+MVC+RIT+LPV | 1 | 0.24 |
| EFV+IDV+RIT | 1 | 0.24 |
| EFV+RIT+LPV | 1 | 0.24 |
| FTC+RAL+DRV+TDF | 1 | 0.24 |
| FTC+RAL+RIT+DRV+TDF | 1 | 0.24 |
| IDV+RIT+LPV | 1 | 0.24 |
| MVC+RIT+DRV | 1 | 0.24 |
| NVP+ATV+RIT | 1 | 0.24 |
| NVP+RIT+LPV | 1 | 0.24 |
| RAL+RIT+SQI+TDF | 1 | 0.24 |
| RIT+LPV | 1 | 0.24 |
| RTF+SQF | 1 | 0.24 |
| 3TC+D4T+NVP+IL2 | 1 | 0.24 |

3TC, lamivudine; ABC, abacavir; ATV, atazanavir; AZT, zidovudine; COB, cobicistat; D4T, stavudine; DDC, zalcitabine; DDI, didanosine; DRV, darunavir; EFV, efavirenz; ETV, etravirine; EVG, elvitegravir; FAP, fosamprenavir; FTC, emtricitabine; HYD, hydroxyurea; IDV, indinavir; IL2, interleukin-2; LPV, lopinavir; MVC, maraviroc; NFV, nelfinavir; NNRTI, non-nucleoside reverse-transcriptase inhibitor; NRTI, nucleoside reverse transcriptase inhibitor; NVP, nevirapine; PI, protease inhibitor; RAL, raltegravir; RCT, randomized controlled trial; RIT, ritonavir; RPV, rilpivirine; RTF, ritonavir full dose; SQF, saquinavir fortovase; SQI, saquinavir invirase; TDF, tenofovir disoproxil fumarate
