# Supplementary material for: Patterns and prognosis of holding regimens for people living with HIV in Asian countries
Source: PLoS One. 2022 Mar 30;17(3):e0264157. doi: 10.1371/journal.pone.0264157 (PMC8967045; doi:10.1371/journal.pone.0264157)
Supplement: S2 Table — Note: ART combinations comprising of less than 5% were grouped as Other. 3TC, lamivudine; ATV/r, atazanavir/ritonavir; AZT, zidovudine; D4T, stavudine; EFV, efavirenz; FTC, emtricitabine; IDV, indinavir; LPV/r, lopinavir/ritonavir; NVP, nevirapine; TDF, tenofovir disoproxil fumarate. (DOCX) [file pone.0264157.s002.docx]

**S2 Table. Patterns of holding regimens by number of previous regimen changes**

| **Number of previous regimen changes** | **ART** | **Number of patients** | **Percent** |
| --- | --- | --- | --- |
| **0** | 3TC+AZT+EFV | 38 | 18.01 |
|  | 3TC+AZT+NVP | 34 | 16.11 |
|  | 3TC+TDF+EFV | 19 | 9.00 |
|  | 3TC+D4T+NVP | 17 | 8.06 |
|  | 3TC+AZT+IDV | 12 | 5.59 |
|  | Other | 91 | 43.13 |
|  | **Total** | **211** | **100** |
| **1** | 3TC+TDF+ATV/r | 26 | 18.44 |
|  | 3TC+TDF+LPV/r | 11 | 7.80 |
|  | TDF+FTC+LPV/r | 10 | 7.09 |
|  | 3TC+TDF+EFV | 9 | 6.38 |
|  | 3TC+AZT+LPV/r | 8 | 5.67 |
|  | Other | 77 | 54.61 |
|  | **Total** | **141** | **100** |
| **>=2** | 3TC+TDF+ATV/r | 6 | 8.22 |
|  | TDF+FTC+LPV/r | 5 | 6.85 |
|  | TDF+FTC+ATV/r | 4 | 5.48 |
|  | Other | 58 | 79.46 |
|  | **Total** | **73** | **100** |

Not: ART combinations comprising of less than 5% were grouped as Other. 3TC, lamivudine; ATV/r, atazanavir/ritonavir; AZT, zidovudine; D4T, stavudine; EFV, efavirenz; FTC, emtricitabine; IDV, indinavir; LPV/r, lopinavir/ritonavir; NVP, nevirapine; TDF, tenofovir disoproxil fumarate
