# Supplementary material for: Patterns and prognosis of holding regimens for people living with HIV in Asian countries
Source: PLoS One. 2022 Mar 30;17(3):e0264157. doi: 10.1371/journal.pone.0264157 (PMC8967045; doi:10.1371/journal.pone.0264157)
Supplement: Attachment — Submitted filename: Second_Response_to_reviewers_Final.docx [file pone.0264157.s004.docx]

Reviewer #2: Many thanks to the authors for addressing my original comments. I have two additional comments:

**Reviewer’s comment #1**

The authors should consider amending the first paragraph of the discussion for better clarity on the holding regimens that were used. The authors note in the introduction that there are several types of holding regimens including lamivudine monotherapy, recycling previously used drugs or adding new ARVs to an existing regimen. It is not clear from the results or the discussion what was the case in this study, although it seems as if patients were simply maintained on their failing regimen.

**Author’s response #1**

Thank you for your comments. We have defined holding regimen in the Methods section as follows:

“We defined PLWH meeting all of the following criteria as taking holding regimens: 1) have initiated cART and been on it forat least 6 months; 2) had virologic failure defined as viral load (VL) ≥1000 copies/mL on two consecutive tests performed within 6 months while on the same regimen; and 3) then remained on the same regimen for at least 6 months after the second VL test. The holding regimen itself can include mono/dual therapy. These definitions applied regardless of the number of drugs that make up the regimen or the category of regimen the subject was on (e.g., 1st, 2nd, or 3rd regimen). Subjects who switched after the first VL test and subjects who switched before 24 weeks after the second VL test were excluded because they did not meet the definition of holding regimen (Fig 1).”

The reviewer is correct in that our definition criteria does not specify that a patient has to be on certain ART combinations to be considered a holding regimen. The patient only has to remain on their failing regimen. We have revised the manuscript for clarification as follows:

**[Introduction]**

When no fully suppressive cART options are available, providers may choose to maintain patients on “holding regimens” in anticipation of future treatments [7]. ~~Several types of holding regimens have been reported, which include lamivudine (3TC) monotherapy, using a greater number of antiretrovirals, and recycling previously used drugs [15-18].~~ The use of holding regimens could have effects on the future immunologic and clinical outcomes of the PLWH.

**[Discussion]**

HIV management has been shifting from prevention of opportunistic infections to managing HIV as a chronic disease with a focus on addressing non-communicable diseases and improving quality of life. However, this paradigm shift is only possible when PLWH are on virally suppressive cART regimens, which may not be achievable when there is drug resistance or limited access to the antiretroviral drug options. This has led to the reliance ~~in some cases~~ on holding regimens in anticipation of future treatments. ~~or extended delays in switching patients on failing regimens.~~ We found that 3TC + AZT + EFV was the most commonly used holding regimen in our Asia regional cohort, followed by 3TC + AZT + NVP.

**Reviewer’s comment #2**

The authors have now added to the definition of a patient taking a holding regimen to include those on mono/dual therapy. Usually patients on these regimens do not have their viral load taken because it is not a virally suppressive regimen. Your definition of a holding regimen includes have 2 consecutive VL>1000 within 6 months on the same regimen. I recommend removing the mono/dual therapy from the definition of holding regimen, unless there were in fact patients who were placed on this regimen.

**Author’s response #2**

Thank you for your comments. As in answer to the previous comment, mono/dual therapy could also be included in the holding regimen only when it meets our study's definition. As shown in the S1 Table, there were several cases where dual therapy was used in our study population. These cases met the definition of a holding regimen which includes 2 consecutive VL>1000 within 6 months on the same regimen. As these cases met the definition of a holding regimen, we included them in our analysis. We revised the sentences to better express that cases that meet the definition of the holding regimen are included regardless of the number of drugs.

**[Materials and methods]**

**Study population**

We defined PLWH meeting all of the following criteria as taking holding regimens: 1) have initiated cART and been on it for at least 6 months; 2) had virologic failure defined as viral load (VL) ≥1000 copies/mL on two consecutive tests performed within 6 months while on the same regimen [22]; and 3) then remained on the same regimen for at least 6 months after the second VL test. The holding regimen itself can include mono/dual therapy. These definitions applied regardless of the number of drugs that make up the regimen or the category of regimen the subject was on (e.g., 1st, 2nd, or 3rd regimen).
